# Melanesian mtDNA Complexity

**DOI:** 10.1371/journal.pone.0000248

**Published:** 2007-02-28

**Authors:** Jonathan S. Friedlaender, Françoise R. Friedlaender, Jason A. Hodgson, Matthew Stoltz, George Koki, Gisele Horvat, Sergey Zhadanov, Theodore G. Schurr, D. Andrew Merriwether

**Affiliations:** 1 Anthropology Department, Temple University, Philadelphia, Pennsylvania, United States of America; 2 Independent Researcher, Philadelphia, Pennsylvania, United States of America; 3 Anthropology Department, Binghamton University, Binghamton, New York, United States of America; 4 Institute for Medical Research, Goroka, Papua New Guinea; 5 Anthropology Department, University of Pennsylvania, Philadephia, Pennsylvania, United States of America; University of Utah, United States of America

## Abstract

Melanesian populations are known for their diversity, but it has been hard to grasp the pattern of the variation or its underlying dynamic. Using 1,223 mitochondrial DNA (mtDNA) sequences from hypervariable regions 1 and 2 (HVR1 and HVR2) from 32 populations, we found the among-group variation is structured by island, island size, and also by language affiliation. The more isolated inland Papuan-speaking groups on the largest islands have the greatest distinctions, while shore dwelling populations are considerably less diverse (at the same time, within-group haplotype diversity is less in the most isolated groups). Persistent differences between shore and inland groups in effective population sizes and marital migration rates probably cause these differences. We also add 16 whole sequences to the Melanesian mtDNA phylogenies. We identify the likely origins of a number of the haplogroups and ancient branches in specific islands, point to some ancient mtDNA connections between Near Oceania and Australia, and show additional Holocene connections between Island Southeast Asia/Taiwan and Island Melanesia with branches of haplogroup E. Coalescence estimates based on synonymous transitions in the coding region suggest an initial settlement and expansion in the region at ∼30–50,000 years before present (YBP), and a second important expansion from Island Southeast Asia/Taiwan during the interval ∼3,500–8,000 YBP. However, there are some important variance components in molecular dating that have been overlooked, and the specific nature of ancestral (maternal) Austronesian influence in this region remains unresolved.

## Introduction

Northern Island Melanesia consists of the two archipelagos just to the east of New Guinea; the Bismarcks and Solomon Islands. Understanding the genetic diversity of its populations is important to prehistoric reconstructions across the Pacific because it was settled by some of the earliest human groups to enter the entire region, and was then the area from which the exploration and colonization of vast stretches of the Pacific commenced at a much later date.

Radiocarbon dates from archaeological sites indicate Northern Island Melanesia was first settled around 40,000 years ago, very soon after people reached the ancient continent of Sahul (present day New Guinea and Australia) [1], [2]. During the next 35,000 years, it remained at a comparatively isolated edge of the human species range. The early populations in Northern Island Melanesia were very small groups of hunter-gatherers. For example, New Ireland, an island over 300 km long, is estimated to have had a pre-Neolithic carrying capacity of less than 1,200 people [3]. While they were dependent on marine resources at first, the people sometimes ventured into the large island interiors [2], [4]. Isolation was not complete. Plant and animal introductions from New Guinea indicate continuing contacts at a very modest level [5]. Short voyages between islands have also been inferred [2], [6], since people had made the windward crossing from New Ireland to Bougainville by 29,000 YBP, and there was a detectable and repeated trickle of New Britain obsidian to New Ireland between 20,000 YBP and ∼7,000 YBP [5]. By extrapolation, movements between this region and as far west as Island Southeast Asia would also have been intermittent.

During the mid-to late-Holocene, at least one significant impulse of influence came from Island Southeast Asia that led to the development of the Lapita Cultural Complex in the Bismarck Archipelago, primarily on its small off-shore islands, at ∼3,300 YBP [7], [8]. A few hundred years after, people bearing the Lapita Cultural Complex had colonized the islands of the Pacific as far east as Tonga and in effect had become the Polynesians (a useful distinction is Remote Oceania, which refers to the Pacific islands beyond the central Solomons settled at ∼3,200 YBP or later, versus Near Oceania, which includes New Guinea and Northern Island Melanesia [9]). In Northern Island Melanesia, variable contacts between the “Lapita People” and the native groups took place along the shorelines, and some later secondary population expansions have been detected in the region as well. As a result of this complex history, Northern Island Melanesian populations are linguistically extraordinarily diverse [10]–[12] as well as genetically heterogeneous.

Here we show through extensive hypervariable region and targeted complete sequencing of mtDNAs that the structure of (maternal) genetic variation in Northern Island Melanesia indicates a long history marked by very small population sizes and limited migration rates until relatively recently. The oldest haplogroups in the region have diversified in such a localized way that particular islands can be identified as their likely homelands. Within islands, the remote inland Papuan speaking groups have diverged the most, and the largest and most rugged islands contain the greatest distinctions among populations. People(s) who entered the region during the Holocene from Island Southeast Asia/Taiwan not only carried the mtDNA “Polynesian Motif” [13], [14] or its precursor, but also branches of haplogroup E. We also identify problems with associating these young haplogroups to the appearance of the Lapita Cultural Complex, or the “Out of Taiwan” model for an Austronesian expansion.

## Results

This section presents the results of our extensive mtDNA survey throughout Near Oceania in the context of the published literature. This includes new whole genome sequences, an analysis of the geographic structure of the patterned variation, and the discussion of the revised estimation of expansion times.

A number of mtDNA haplogroups common in Near Oceania have not been found west of New Guinea (i.e., macrohaplogroups M27 and M29, and with some rare exceptions, P, Q, and M28 [15], [16]). On the other hand, many haplogroups present in Southeast Asia are missing east of the Wallace Line (most branches of M, as well as B4c, B5, C, D, G, and U). This pattern reflects the long isolation of the populations that entered Near Oceania. Two younger mtDNA lineages do occur in appreciable frequencies in both regions, namely B4a1a1 and branches of E.

### Ancient Near Oceania haplogroups

#### Haplogroup P

**Figure 1:**
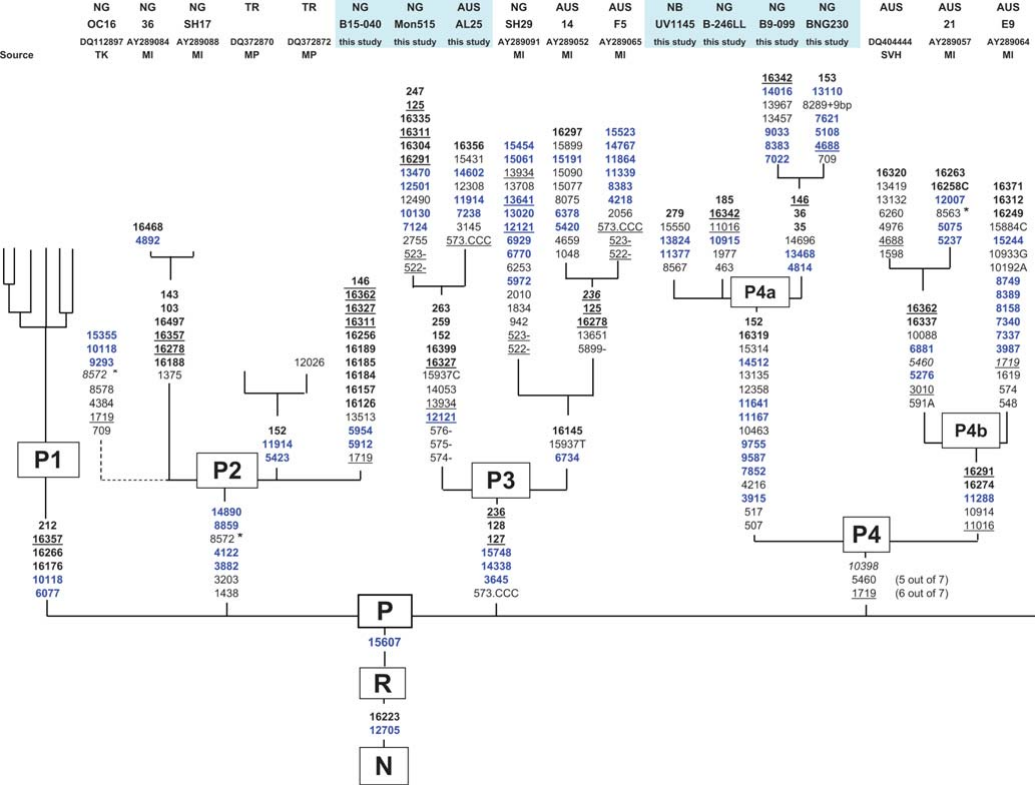
Haplogroup P phylogeny for Near Oceania (branches shared with Australian Aborigines also shown). The branches of P found only in Australian Aborigines, and details of the P1 branches, are available in [17], supplementary materials. Control region mutations are in bold, those that recur in this phylogeny are underlined, those in blue are synonymous transitions, and transversions are noted with a base suffix. Asterisks denote substitutions that can be both synonymous and nonsynomous because of gene overlap (nts 8563 and 8572). These were regarded as nonsynomous. The dotted line in the tree denotes a missing control region sequence. The poly C regions in HVS1 and 2 as well as 16519 are excluded. Proveniences are listed at the top, abbreviated as follows: NG–New Guinea, TR–Trobriands, AUS–Australian Aborigine, NB-New Britain. Sample numbers, GenBank accession numbers, and sources are listed underneath. Source abbreviations are: SV–[18]; TK–[25]; MI–[19]; MP–[33].

Haplogroup P is the oldest branch of macrohaplogroup R in the region. Figure 1 shows the different known branches of P in Melanesia and their defining mutations (table S1 gives further details). Source references for the different branches, including the current study, are at the top of the figure. The branching of P1 is abbreviated since it has been detailed elsewhere [17]. The branching pattern at the base of P4 is ambiguous due to the apparent occurrence of back mutations at nucleotide sites (nts) 1719 and 5460. We have identified new branches of P2, P3 and P4. P3 and probably P4 retain old connections between Near Oceania and Australia, but branch P4a appears to be specific to Near Oceania, and branch P4b appears to be limited to Aboriginal Australia.

In addition to these two ancient connections between Australia and Near Oceania, there is a possible third one, inferred from shared HVS1 transitions at 16184, 16223, 16256, and 16519. In Australia, this haplotype has been called haplogroup N(S) or AuA (Genbank AF346965) [18], [19] and in New Guinea it was referred to as an unnamed branch under “cluster II” (Genbank AB119390, AB119397, AB119411, AB119420 and AB119397) [20]. It may also have been found in shorter Australian HVS1 sequences, as in Genbank AF176175 [21] and “sample number 13.1”[22].

The estimates for the Time to Most Recent Common Ancestor (TMRCA) for the P branches are in table 1, using the ***ρ*** technique [23] and two different mutation rates [24], [25]. The rankings of the ***ρ*** values and TMRCAs are the same for both techniques, i.e., P3>P1>P4a>P2, and the general conclusion is the same–that their founder ages in Near Oceania date to ∼30–50,000 YBP.

**Table 1:** Coding region age estimates for Haplogroups common in Near Oceania

			Coding Region nt577-nt16022 a						Synonymous Transitions b					
Haplogroup		*n*	ρ	σ	TMRCA, years	SD	Founder age, years	SD	ρ	σ	TMRCA, years	SD	Founder age, years	SD
P1		6	8.33	1.35	42,800	7,000	53,000	10,000	4.50	0.96	30,400	6,500	44,000	11,600
P2		6	3.17	0.50	16,300	2,600	47,100	12,800	1.67	0.33	11,300	2,300	38,300	13,700
P3		5	11.60	1.81	59,600	9,200	75,000	12,800	5.60	1.20	39,200	8,200	59,500	14,300
P4a		4	6.25	1.39	32,100	7,200			3.75	1.10	25,400	7,400		
M27		7	13.43	2.18	69,000	11,200	84,400	14,300	5.86	1.45	39,600	9,800	53,100	13,700
M28		8	6.25	1.37	32,100	7,000	57,800	13,500	3.00	0.97	20,300	6,500	47,300	15,000
	M28a	6	3.33	0.88	17,100	4,500			1.67	0.67	11,300	4,500		
	M28b	2	8.00	2.00	41,000	10,300			4.00	1.41	27,000	9,600		
M29		4	5.00	1.87	25,700	9,600	56,500	15,800	3.00	1.52	22,000	10,300	42,300	16,000
Q		23	8.65	1.38	44,500	7,100	70,200	13,500	4.91	1.02	33,200	6,900	53,500	13,600
	Q1&Q2	16	7.69	1.49	39,500	7,700			5.19	1.87	35,100	9,200		
	Q1	13	5.23	1.01	26,900	5,200			3.23	0.82	21,900	5,500		
	Q2	3	8.67	2.36	44,500	12,100			5.00	1.73	33,800	11,700		
	Q3	7	5.57	0.83	28,600	4,300			3.29	0.64	22,200	4,300		
	E1a^c^	2	1.00	0.71	5,100	3,600			0.50	0.50	3,400	3,400		
	E1b^c^	3	1.50	0.58	5,100	3,000			1.00	0.58	6,800	3,900		
B “Pol.Motif” d		13	1.54	0.34	7,900	1,700			0.92	0.27	6,200	1,800		

a One substitution per 5,139 years (Mishmar et al. 2003).

b One synonymous transition per 6,764 years (Kivisild et al. 2006)

c Island Melanesia only

d Source: Pierson et al. (2006)

Table S2 gives the distribution of the major haplogroups in our series. P has its highest frequency in New Guinea and P1, its most common branch, has its highest concentration and greatest diversity in the highlands. P2 and P4 are also more common in New Guinea than elsewhere.

#### Macrohaplogroup M

Many deep branches of M have been found throughout Asia, especially India [26]–[32]. Pierson et al. [33] showed that all known branches of M diverged separately from the base, with the possible exception of Melanesian M29 and Q which may be somewhat more closely related.

**Figure 2:**
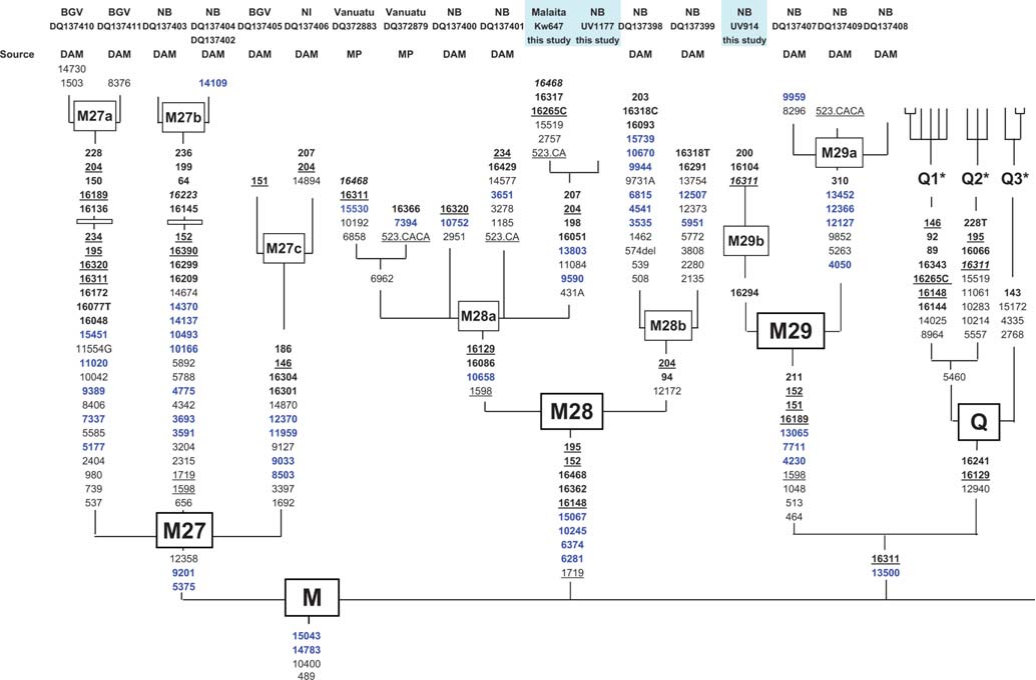
Upper Pleistocene macrohaplogroup M phylogeny for Near Oceania (haplogroup E is excluded). Abbreviations follow figure 1. Additional sample abbreviations are BGV–Bougainville, and NI-New Ireland. Additional source abbreviation is DAM–[36]. Boxes on M27a and b indicate inferred additional branches defined by control region sequences. The complete Q tree is presented in figure 4.

Figure 2 shows the main branches of macrohaplogroup M that occur in Near Oceania, including new branches of M28 and M29 identified in this study. To date, there are no established links between Aboriginal Australia and Near Oceania within any M haplogroup. As with P, the Near Oceanic branches of M apparently developed around the time of initial settlement beginning before ∼30,000 years ago [current study, 17,19,34–37]. The TMRCAs in table 1 for these Near Oceanic M haplogroups and their branches suggest many are as old as those for P.

**Figure 3:**
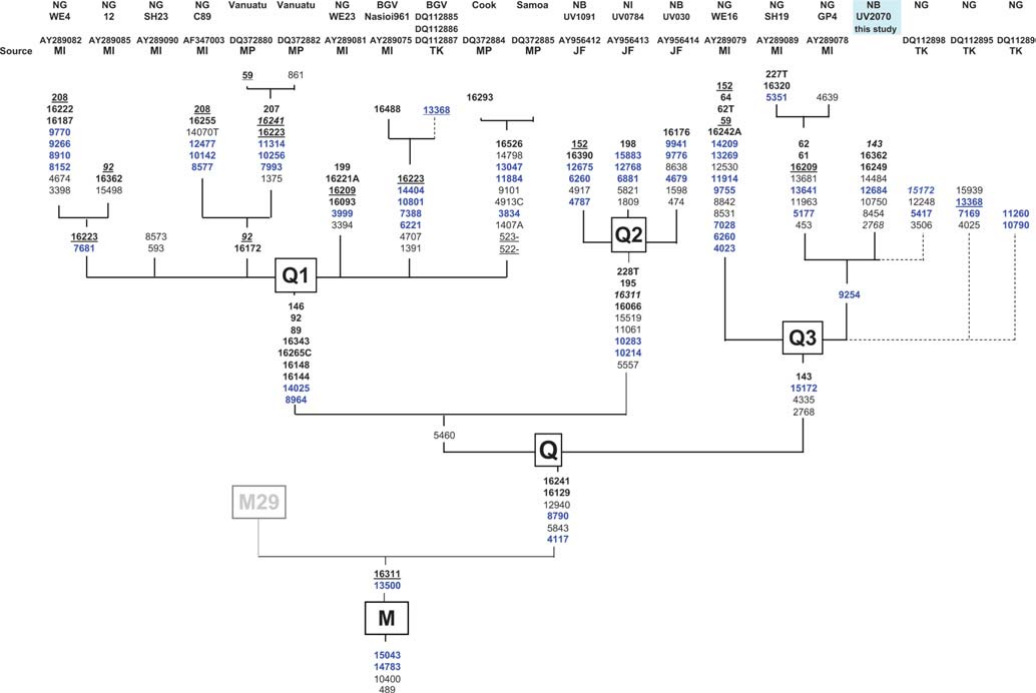
Haplogroup Q phylogeny. Abbreviations follow figure 1. Additional source abbreviation is JSF–[35].

Haplogroup Q is the most common Near Oceanic subdivision of M (see tables S1 and S2). Q has a large number of defining mutations at its base and long internal branches (figure 3). The Q1 branch is especially common in West New Guinea, in the Markham Valley, throughout New Britain, and north Bougainville. Q2 is most common among certain inland Papuan groups of New Britain (Baining and Ata). Although we cannot be certain Q2 originated here, it clearly underwent an expansion among the inland Papuan groups of New Britain. Both Q1 and Q2 have been found as far to the east as Fiji [38]. We could identify only seven Q3 samples: two from the New Guinea highlands [also reported in 19,21] and five from West New Britain.

Other deep branches of macrohaplogroup M probably developed in Northern Island Melanesia (table S2) since they are most common and diverse there. Haplogroup M29 (possibly related to Q–see figure 2) is most common in East New Britain. M27 is centered in Bougainville, with the M27a branch common in north Bougainville, M27b most common in east New Britain, while M27c is more scattered. Haplogroup M28 is relatively common, and a total of 8 M28 samples have been completely sequenced. More than 80% of our M28 samples were from New Britain, and its greatest diversity is there. The center of M28 diversity is among the Baining and Ata in East New Britain. While M28 is relatively rare in New Ireland, Bougainville, and the central Solomons, we found it at fairly high frequencies in some Remote Oceanic populations-Santa Cruz (29%), Vanuatu (30% of our series, and about the same in [38]), and also in New Caledonia, Fiji, and rarely in Polynesia [16], [20]. This M28 distribution suggests a portion of Remote Oceanic and Polynesian mtDNA comes from a New Britain (and Papuan) origin.

### Young haplogroups

As mentioned, a second set of mtDNA haplogroups is found in both Island Southeast Asia and Oceania and dates to the Holocene.

#### Haplogroup B4a1a1

**Figure 4:**
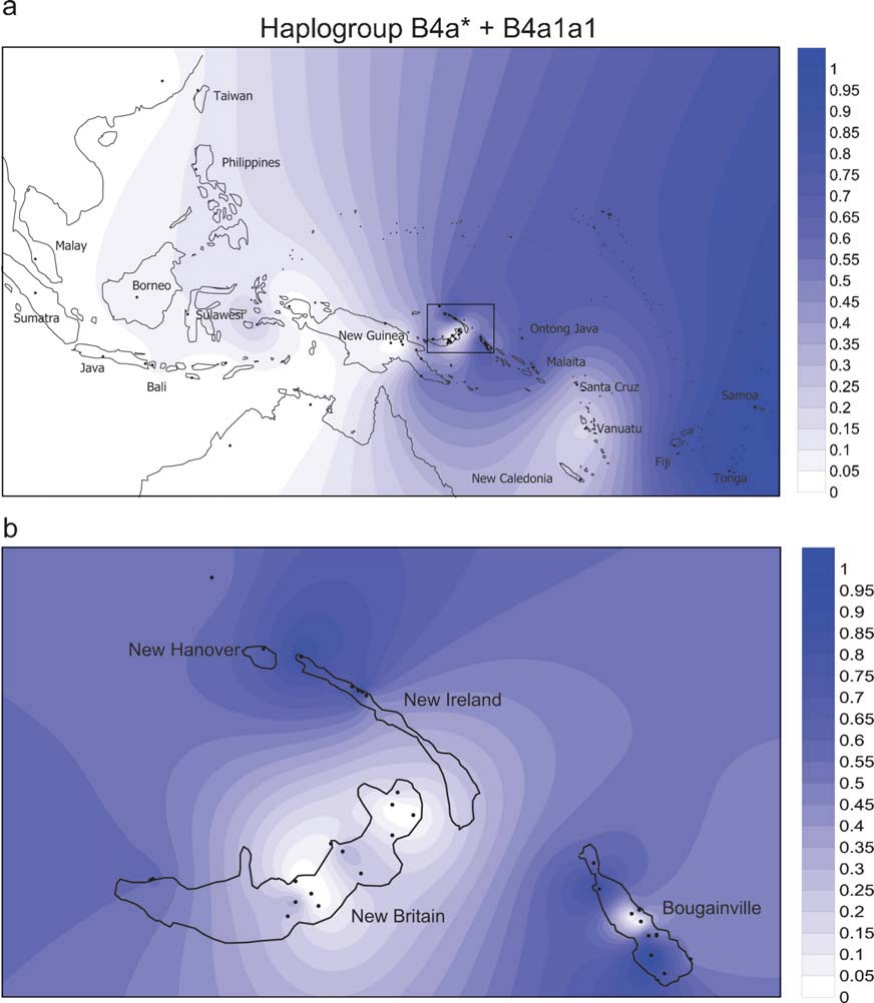
Spatial frequency distribution of haplogroup B4a* and B4a1a1 in Island Southeast Asia and the western Pacific, created using the Kriging algorithm of the Surfer package of haplogroups. Figure 4b presents the detailed distribution for Northern Island Melanesia. Data details are provided in table S3.

Almost 40% of our samples are B4a1a1, which includes the so-called “Polynesian Motif” (table S2). This has been tied to an Austronesian expansion out of Taiwan that led to the development of the Lapita Cultural Complex in the Bismarcks, and finally to the settlement of Polynesia and Micronesia [13], [33], [39]–[41]. This association depended heavily on the haplogroup distribution. The “Motif” is very common in Polynesia, Micronesia, and many parts of Near Oceania, and is absent in the Papuan New Guinea highlands [13], as well as in some Papuan-speaking areas of Northern Island Melanesia (tables S2, S3, and figure 4a). The “Motif” has also been confirmed in central and eastern Indonesian populations in low frequencies [42], and it could have originated either there or in Near Oceania [see 33]. It was also carried to Madagascar [43]. Whole mtDNA sequencing has identified the immediate precursor to the “Motif” in Taiwan Aboriginal groups [40], apparently strengthening the “Out of Taiwan” hypothesis (N.B. the key difference between this precursor and the “Motif” is the transition at nts 14022; the transition at 16247 had been used to identify the “Motif” in many earlier studies, but it is hypermutable in our series and therefore is not reliable).

However, this association has its problems. As shown in figure 4a and 4b, haplogroup B4a1a1 is rare in Island Southeast Asia and is not particularly common in the New Britain vicinity, which is at the center of early Lapita sites (they are mostly on nearby small islands) [5]. The “Motif” becomes very common and almost reaches fixation in some New Ireland and Bougainville groups, but some of these groups speak Papuan-languages [44]. The “Motif” is also low in frequency in Vanuatu (an area of Remote Oceania settled first by Lapita people [5]), as well as Fiji, before it reaches high frequencies again in Polynesia. Therefore, the distribution association is not so compelling as it once appeared. Also, the distribution of the “Motif” precursor is poorly understood. It could be considerably more widespread than Taiwan because its identification relies on sequencing nts 14022, which has not been generally done.

The TMRCA for both the precursor of the “Motif” in Taiwan (B4a1a), and the “Motif” in Near Oceania are also troublesome for the genetic part of the “Out of Taiwan” hypothesis. The molecular estimates are older than the corresponding archaeological dates. The Neolithic period in Taiwan that could have led to a subsequent Austronesian expansion dates only to about 6,000 years ago [8], while the best coalescence date for the precursor B4a1a in Taiwan was estimated at 13,000±3,800 YBP [40]. In the Bismarck Archipelago, the Lapita cultural complex dates to no earlier than 3,300 YBP [5], but the TMRCA for the “Motif” in Papuans and Polynesians was 9,300±2,000 YBP (since then, the date for the “Motif” been estimated for 13 sequences [33], using two different methods, at 7,900±1700 YBP, and 6,200±1800 YBP). However, as covered in the Discussion, the variances of these coalescence estimates are greater than generally acknowledged, so that an accommodation with the archaeological dates remains a possibility.

#### Haplogroup E

This relatively uncommon haplogroup, a subdivision of M9, was thought to be limited to Mainland and Island Southeast Asia [45]. Its E1a branch had been found in Thailand, in Sabah Aborigines, in Taiwan Aborigines, as well as across Indonesia. The E1b branch had been found in Indonesia and the Philippines (apparently absent in Taiwan). The rarer E2 had only been found in Taiwan Aborigines and Filipinos.

**Figure 5:**
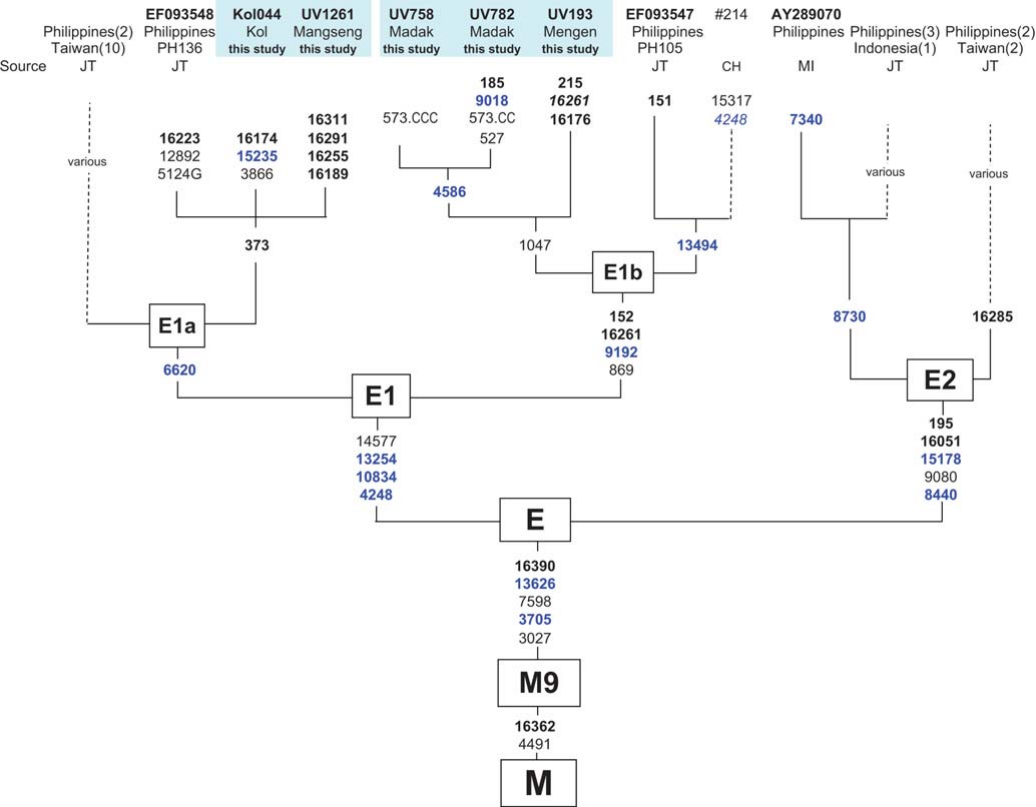
Haplogroup E phylogeny. Abbreviations follow figure 1. Additional source abbreviations are CH–[67] and JT-[68].

We have added 5 complete E sequences now identified in Northern Island Melanesia (figure 5). The Island Melanesian E1a branches share a key mutation with one from the Philippines (nts 373), and the Melanesian E1b branches share 4 mutations with another branch from the Philippines.

**Figure 6:**
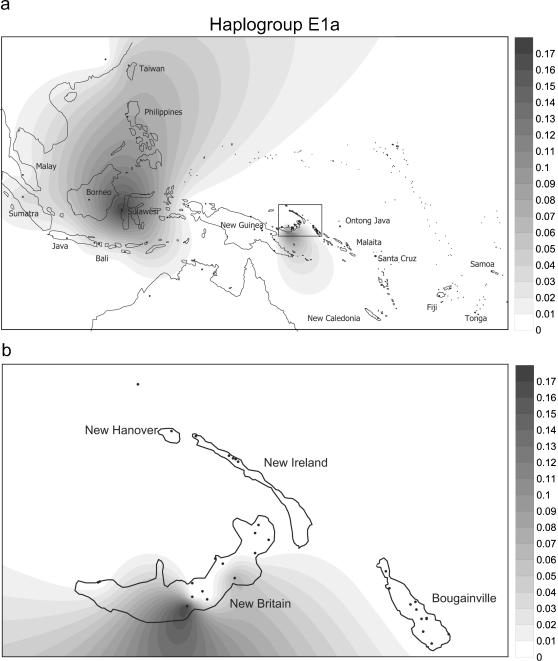
Spatial frequency distribution of haplogroup E1a in Island Southeast Asia and the western Pacific (6a), and details for Northern Island Melanesia (6b) created using the Kriging algorithm of the Surfer package of haplogroups. Data details and references are provided in table S3.

**Figure 7:**
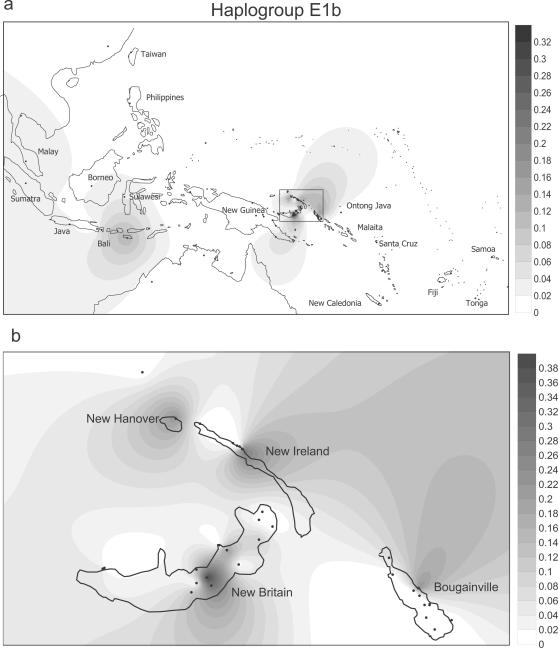
Spatial frequency distribution of haplogroup E1b in Island Southeast Asia and the western Pacific (7a), and details for Northern Island Melanesia (7b). Data details and references are provided in table S3.

The distribution of E in our series was spotty (table S2). 75 samples were E1b, and most of these were from New Britain (the Papuan-speaking Ata and Sulka). The rest were scattered across a number of Oceanic speaking groups in the region. We have not been able to ascertain from the literature if E was dispersed to Remote Oceania, since its identification depends on sequencing nts 16390 in HVS1. Figures 6 and 7 give a sense of the heterogeneous distributions of E1a and E1b in Island Melanesia and the Southwest Pacific as currently understood (table S3 has the data references). We also found two E2s, which have been identified in Taiwan as well as Indonesia and the Philippines.

In sum, E is a second young haplogroup that was brought to Near Oceania, specifically to New Britain, from the west. Coalescence time estimates for E1a and E1b in our series suggest both branches are about the same age as the “Polynesian Motif,” or slightly younger (table 1). The distribution of E1b suggests a connection between Island Southeast Asia (excluding Taiwan) and Northern Island Melanesia.

The distributions in Near Oceania of the haplogroups B4b1, F, M7, and Y are too rare to be informative.

### Analysis of molecular variance

**Figure 8:**
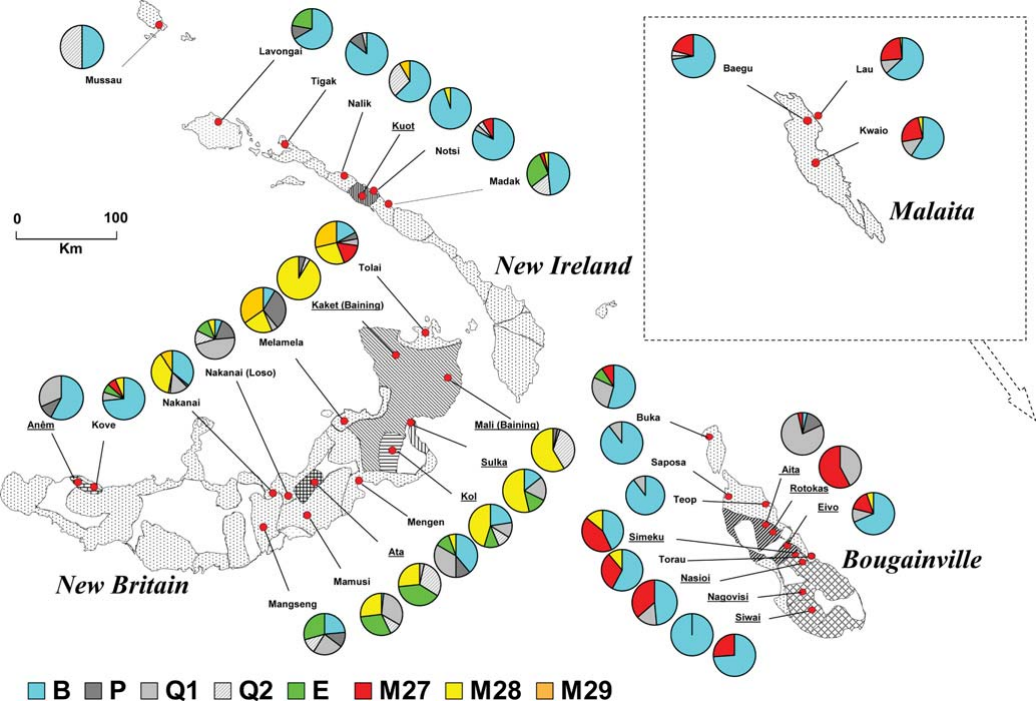
Northern Island Melanesian Southwest mtDNA haplogroup frequency distribution taken from our series (table S2).

Figure 8 gives a sense of the haplogroup variation in the core region of Northern Island Melanesia (table S2 shows the actual haplogroup incidences). While there is an island-by-island distinction, New Britain is considerably more internally diverse than Bougainville, with both New Ireland and Malaita considerably less so.

An analysis of molecular variance (AMOVA) was performed on the HVS1 and HVS2 sequences to quantify the mtDNA population structure (table 2). Only 32 populations from the 4 largest islands in our Island Melanesian series were included, since these were from the most intensively sampled area and could be used to compare population variation within-and among-islands, as well as within-and among-language groups. The among-population variance represented a very large proportion of the total–almost 28%, reflecting the remarkable population structure in this small region of the Southwestern Pacific. Although comparing AMOVA results across studies is not straight-forward, this value for these 4 islands is unsurpassed in the mtDNA literature for among-population variation within entire continents. In a global survey, the general among-group, within-continent variance proportion for the mtDNA control region was reported as ∼8% [46], and the most recent report on African among-group mtDNA variance components is ∼20% [47]. Over the entirety of North and South America, where native populations have undergone extreme drift, the estimate of the total among-group mtDNA variance is 26% [48], close to the estimate for Northern Island Melanesian populations. This indication of very high mtDNA among-group variation is no aberration. A global survey of short tandem repeat polymorphism variation also suggested very high Melanesian diversity, even though only two Melanesian population samples were included [49].

**Table 2:** AMOVA based on mtDNA HVS1 and HVS2

				Variance Components (%)		
Grouping	*n*	No. of Populations	No. of Groups	Between Groups	Within Groups	Within Populations
No grouping (32 populations)	1223	32	1	…	27.5	72.5
Geography (4 Islands)^ a^	1223	32	4	12.2	17.8	70
Language (2 groups)^ b^	1223	32	2	*2.9* *	25.6	71.4
Oceanic (20 populations)	749	20	1	…	14.9	85.1
Papuan (12 populations)	474	12	1	…	40.4	59.6

a Groups for geography: New Ireland, New Britain, Bougainville and Malaita

b Linguistic groups: Papuans, Oceanic

* p = 0.07

Partitioning the variance showed that while the variation among the four island groups was significant (12%), the variation among-populations within-islands was even greater (17.8%). New Britain, the largest and most rugged island in our series, contributed disproportionately to the within-island variance component, and New Ireland, which is over 300 kilometers long but averages less than 10 kilometers in width along most of its length, contributed the least. The size and topographical complexity of the islands is related to the genetic diversity of their populations.

Within-population mtDNA diversity across the region is related to the same pattern affected by population size and isolation. As shown in table S4, the smallest population haplotype diversity values are for seven inland Papuan groups on different large islands (the Mali, Kaket, Anêm, Ata, Aita, Rotokas, and Nagovisi), while 2/3 of the highest haplotype diversity values are for beach-dwelling Oceanic groups. By island, the average population haplotype diversities are lowest for New Britain and highest for New Ireland.

Partitioning the variance by the two major language families (Oceanic vs. Papuan) produced a non-significant between-group statistic (2.9%). This is not surprising since languages belonging to these families are spoken on different islands. However, the variation among Papuan-speaking groups (40.4%) was far greater than among Oceanic-speaking groups (14.9%)–an important distinction, since the Oceanic-speaking groups tend to be distributed along the coastlines, and they were introduced much more recently in the region.

### Multidimensional Scaling Plot (MDS)

**Figure 9:**
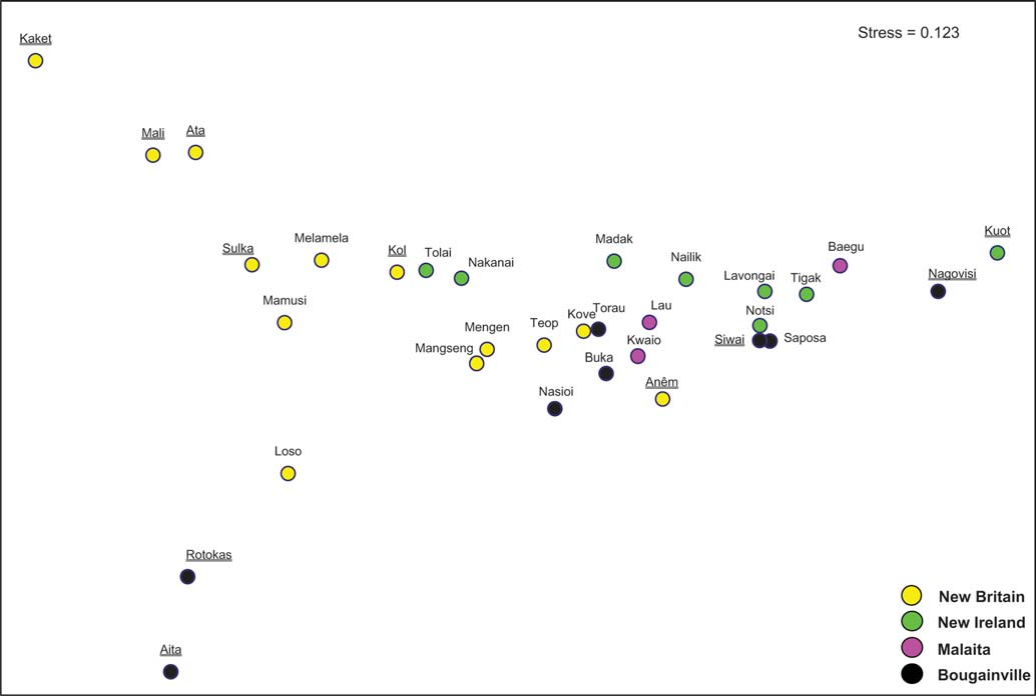
Two-dimensional multidimensional scaling plot, generated from pairwise Fst values of mtDNA variation in Northern Island Melanesian populations. First axis is horizontal, second is vertical. Papuan speaking populations are underlined.

To visualize the population relationships, we performed a non-parametric multidimensional scaling (MDS) on the pairwise F_ST_ statistics (figure 9). This plot has a stress value of 0.123 with an r^2^ of 0.95, and therefore is a good representation of the population distinctions. The general island-by-island clustering of the populations is apparent (with New Britain populations clustered to the left top quadrant, New Ireland to the upper right, and Bougainville populations generally in the bottom half). The extreme outliers are Papuan-speaking groups, consistent with the AMOVA results. The New Britain Ata, Mali, and Kaket form one extreme cluster that contrasts with the New Ireland Kuot and south Bougainville Nagovisi at the other extreme of the first dimension; the second dimension contrasts the same New Britain Papuan cluster with the north Bougainville Aita and Rotokas. The same populations have the lowest haplotype diversities (table S4). However the Anêm, who are Papuan-speaking, fall towards the middle of the distribution. Since they show signs of substantial linguistic borrowing from their Oceanic-speaking neighbors on the shore, the Kove [50], this is not surprising.

In order to identify important associations, we correlated the population scores on the two MDS dimensions with the population haplogroup frequencies. The first dimension was negatively correlated with the frequency of haplogroup B4a1a1 (r = −0.95) and positively correlated with frequencies of M28 (r = 0.70). This explains the contrast between the New Britain and Kuot/Nagovisi Papuan-speakers. Population scores on the second dimension are most strongly correlated with Q1 frequencies (r = 0.83), contrasting the Aita and Rotokas with the Baining and Ata cluster. The next strongest correlations for the second dimension scores are with M27 (r = 0.50) and M28 (r = −0.49).

## Discussion

This intensive sampling in the major islands of Northern Island Melanesia, combined with sequencing of HVS1, HVS2, and whole mtDNA genome sequencing for ambiguous haplogroups, indicates a remarkably structured pattern of population diversity. It is the difference among the remote inland Papuan-speaking clusters on separate islands that drives the pattern of overall mitochondrial variation across the region, while the coastal groups are more intermixed. This is apparent from the MDS plot of population distances and from the AMOVA results. The shoreline Oceanic speaking groups do have island-by-island distinctions, but much less than the Papuan isolates. This overall pattern has very likely been caused by differences between the shore and inland groups in effective population sizes and marital migration rates [35], [51]. The larger and the more rugged the island, the more apparent is this “beach vs. bush” distinction. As mentioned, the islands of Remote Oceania are all considerably smaller and do not appear to have retained the same levels of among group diversity across whole archipelagos. Their within population haplotype diversities are also considerably lower [16], since the frequency of the full “Motif” in the central Pacific is very high.

In addition, the phylogeography of the ancient set of haplogroups suggests the ancient haplogroup variants originated in different Papuan-speaking areas and have tended to survive there. Taken together, while associating haplogroup trees and their TMRCAs to particular population histories is a complex endeavor (see [52] for a complex Pacific example), at least in this region it produces reasonable results that are also compatible with AMOVA and population distance analyses.

Molecular dating estimates remain approximations and should not be used alone to reject one prehistoric migration scenario in favor of another. The mtDNA methodology is being revised yet again [see, among others, 25,53–56]. At the moment, the most attractive technique focuses on the accumulation of synonymous transitions in the coding region [25], with an estimated average rate at 3.5×10^−8^ per year (S.D. 0.1×10^−8^), using a human-chimpanzee split time and TMRCA date of 6 and 6.5 million years ago, respectively, for calibration. These are the same calibration dates that have been used before (see [24], citing [57]). However, these point estimates are less precise than acknowledged. The range of legitimate estimates for the human-chimpanzee split from the fossil record is from 4.98 to 7.2 million years, and for any of these estimates there is a 95% confidence interval of −12% to+19% [58]. The earliest fossil evidence for hominid erect bipedalism is *A. anamensis* at ∼4 million years, providing an unequivocal lower bound for the split. If the 4.98 million year split is used rather than the one at 6 million, all resulting age estimates will be 17% less, and there will be the additional variance component contributed by the 95% confidence interval. For example, an estimated TMRCA of 6,000 YBP (as with the current estimate for the “Polynesian Motif” in table 1) could be more than 1,000 years too early or too late, with an uncertainty of ∼10–15% added to the estimate and standard deviation. The same percentages would apply to the much older estimated TMRCA ages for the most ancient haplogroups as well.

Tying the B4a1a1 haplogroup, which predominates in Polynesia and Micronesia, with mid-Holocene population movements originating in Taiwan [59] or Wallacea [60] has gotten more complicated as coverage has improved [45], [51]. What is clear is that precursors of the “Motif” originated to the west of Wallacea in the early Holocene; that the full “Motif” with the transition at 14022 developed in eastern Island Southeast Asia or Near Oceania; that its frequency varies a great deal across Island Southeast Asia, Near Oceania, and sections of Remote Oceania before becoming very common in central Polynesia; and that subtypes of haplogroup E, which also developed in Island Southeast Asia during the Holocene, also have a very spotty distribution and were carried to the Bismarcks but probably no further east. Also, the analyses of skeletal remains associated with Lapita or earliest Polynesian sites have still not yielded any B4a haplogroup identifications [61], [62]. It must be remembered that mtDNA haplogroups (along with Y variants) are especially affected by genetic drift, since the effective sample size is only ¼ that of an autosomal marker in any population. Also, the small populations colonizing Near and Remote Oceania were probably subject to exaggerated drift distortions [53], [56].

The main point is that mtDNA variation among populations in Northern Island Melanesia is extreme but understandable. It reflects the very ancient settlement of the region; the subsequent isolation and drift of many inland populations; some subsequent internal population expansions; the introduction of two haplogroups and populations in the mid Holocene, combined with some intermixture among many groups, especially those living along the shorelines. Because the mtDNA only reflects a very small (exclusively maternal) fraction of the heritage of an individual or population, it may yield a biased result, but this survey also shows its power in elucidating ancient population dynamics.

## Materials and Methods

The samples analyzed were selected from our Southwest Pacific collection. Its core consisted of blood samples collected in three recent field seasons in the Bismarck Archipelago. This primary set was augmented with plasmas and urines from older collections, described elsewhere [17]. Information on survey subjects included their language, a short genealogy, current residence, and familial birthplaces (used to assign location), although such details were not available from some of the other collections. The primary samples were collected, and all selected samples were analyzed, with informed consent protocols approved by the appropriate Human Subjects Ethical Committees of Papua New Guinea, the University of Michigan, Binghamton University, and Temple University.

One sample from each identified matriline was selected for the initial mtDNA control region analysis. The analysis of the samples occurred in three phases: (1) sequencing of hypervariable segments 1 and 2 (HVS1 and HVS2); (2) for those samples not definitely assigned to a known haplogroup on this basis, RFLP screening for the two mutations defining macrohaplogroup M (DdeI 10394, AluI 10397) and, depending on the presence or absence of these, additional RFLPs known to identify other haplogroups in the Southwest Pacific [37], [63], [64]; and (3) sequencing of the coding region on 16 representative samples from the remaining major haplogroups that could not be assigned to currently published sublineages.

DNA was extracted from 100 to 200 µl of buffy coat, plasma, or urine (depending on the source of the sample) by using either the guanidine-silica based IsoQuick extraction kit (Orca Scientific, Bothell WA) or the column-based Qiagen extraction kit (Qiagen, Valencia CA). In preparation for sequencing, the mtDNAs were PCR amplified following standard protocols, and employing Platinum Taq Polymerase (Invitrogen, Carlsbad CA). The control region was amplified using primers spanning nps 15938 to 00429. The coding region was amplified using the PCR primers and conditions of Rieder et al. [65]. Successful amplification was verified by electrophoresis on 1% ethidium bromide stained agarose gels. Samples were prepared for sequencing by an ExoI digest followed by filtration through a Millipore 96-well filter plate (Millipore, Billerica MA) to remove single stranded DNA and unincorporated nucleotides. PCR products were sequenced using various versions of the BigDye Terminator Sequencing kits from ABI (Applied Biosystems Inc) on an ABI 377XL automated sequencer using conditions described previously [66]. Custom designed internal sequencing primers were used for all large PCR fragments to increase double-fold coverage.

Contig assemblage and sequence alignment was accomplished with Sequencher: Forensic Version 4.1.9 (GeneCodes, Ann Arbor MI). Sequencher was also used to determine the synonymous transitions. The phylogenetic tree was inferred from median-joining networks rooted to L3.The tree was hand-checked to resolve several homoplasies. A few ambiguities remained, and we tended to be conservative in interpreting those cases.

### Accession numbers

The GenBank accession numbers (http://www.ebi.ac.uk/embl/index.html) for the 16 complete mtDNA sequences are EF061145-EF061159.

## Supporting Information

Table S1 Defining Mutations for mtDNA Haplogroups found in Island Melanesia
(0.02 MB XLS)

Table S2 mtDNA Lineage Occurences in Island Melanesia (Individuals)
(0.03 MB XLS)

Table S3 Longitudes, Latitudes and Frequencies used for Figures 6 & 7
(0.02 MB XLS)

Table S4 Measures of Haplotype diversity in 32 Northern Melanesia population using HVS1
(0.02 MB XLS)

## References

[1] Leavesley M, Chappell J (2004). Buang Merabak: additional early radiocarbon evidence of the colonisation of the Bismarck Archipelago, Papua New Guinea.. Antiquity Project Gallery.

[2] Allen J, Sand C (2003). Discovering the Pleistocene in Island Melanesia.. Pacific Archaeology: Assessments and Anniversary of the First Lapita Excavation (July 1952) Koné, Nouméa, 2002.

[3] Summerhayes GR, Chiu S, Sand C (2007). The rise and transformations of Lapita in the Bismarck Archipelago.. From Southeast Asia to the Pacific: Archaeological Perspectives on the Austronesian Expansion and the Lapita Cultural Complex.

[4] Pavlides C, Gosden C (1994). 35,000 year-old sites in the rainforests of West New Britain, Papua New Guinea.. Antiquity.

[5] Summerhayes GR, Friedlaender JS (2007). Island Melanesian Pasts-A View From Archaeology.. Genes, Language, and Culture History in the Southwest Pacific.

[6] Anderson A, O'Connor S, Veith P (2000). Slow boats from China: Issues in the prehistory of Indo-Pacific seafaring.. East of Wallace's Line: Studies of Past and Present Maritime Cultures of the Indo-Pacific Region Modern Quaternary Research in Southeast Asia.

[7] Spriggs MJT (1997). The Island Melanesians.

[8] Bellwood P, Harris DR (1996). The origins and spread of agriculture in the Indo-Pacific region: Gradualism and diffusion or revolution and colonization?. The Origin and Spread of Agriculture and Pastoralism in Eurasia.

[9] Green RC, Pawley A (1991). Near and Remote Oceania-disestablishing “Melanesia” in culture history.. Man and a half: Essays in Pacific anthropology and ethnobiology in honour of Ralph Bulmer.

[10] Dunn M, Terrill A, Reesink G (2002). The East Papuan languages: A preliminary typological appraisal.. Oceanic Linguistics.

[11] Lindström E, Terrill A, Reesink G, Dunn M, Friedlaender JS (2007). The languages of Island Melanesia.. Genes, Language, and Culture History in the Southwest Pacific.

[12] Ross M (1988). Proto Oceanic and the Austronesian Languages of Western Melanesia.

[13] Redd AJ, Takezaki N, Sherry ST, McGarvey ST, Sofro ASM (1995). Evolutionary History of the COII/tRNALys Intergenic 9 Base Pair Deletion in Human Mitochondrial DNAs from the Pacific.. Mol Biol Evol.

[14] Hertzberg M, Mickleson KNP, Serjeantson SW, Prior JF, Trent RJ (1989). An Asian specific 9-bp deletion of mitochondrial DNA is frequently found in Polynesians.. Am J Hum Genet.

[15] Hill AVS, Bowden DK, Trent RJ, Higgs DR, Oppenheimer S (1985). Melanesians and Polynesians share a unique alpha-thalassemia mutation.. Am J Hum Genet.

[16] Kayser M, Brauer S, Cordaux R, Casto A, Lao O (2006). Melanesian and Asian Origins of Polynesians: mtDNA and Y-chromosome Gradients Across the Pacific.. Mol Biol Evol.

[17] Friedlaender JS, Schurr TS, Gentz F, Koki G, Friedlaender F (2005). Expanding Southwest Pacific mitochondrial haplogroups P and Q.. Mol Biol Evol.

[18] van Holst Pellekaan SM, Ingman M, Roberts-Thomson J, Harding RM (2006). Mitochondrial genomics identifies major haplogroups in Aboriginal Australians.. Am J Phys Anthropol.

[19] Ingman M, Gyllensten U (2003). Mitochondrial genome variation and evolutionary history of Australian and New Guinean aborigines.. Genome Research.

[20] Ohashi J, Naka I, Tokunaga K, Inaoka T, Ataka Y (2006). Brief communication: mitochondrial DNA variation suggests extensive gene flow from Polynesian ancestors to indigenous Melanesians in the northwestern Bismarck Archipelago.. Am J Phys Anthropol.

[21] Redd AJ, Stoneking M (1999). Peopling of Sahul: mtDNA variation in Aboriginal Australian and Papua New Guinean populations.. Am J Hum Genet.

[22] Lum JK, Rickards O, Ching C, Cann RL (1994). Polynesian mitochondrial DNAs reveal three deep maternal lineage clusters.. Hum Biol.

[23] Saillard J, Forster P, Lynnerup N, Bandelt H-J, Norby S (2000). mtDNA variation among Greenland Eskimos: the edge of the Beringian expansion.. Am J Hum Genet.

[24] Mishmar D, Ruiz-Pesini E, Golik P, Macaulay V, Clark AG (2003). Natural selection shaped regional mtDNA variation in humans.. Proc Natl Acad Sci U S A.

[25] Kivisild T, Shen P, Wall DP, Do B, Sung R (2006). The role of selection in the evolution of human mitochondrial genomes.. Genetics.

[26] Metspalu M, Kivisild T, Metspalu E, Parik J, Hudjashov G (2005). Most of the extant mtDNA boundaries in South and Southwest Asia were likely shaped during the initial settlement of Eurasia by anatomically modern humans.. BMC Genet.

[27] Kivisild T, Tolk HV, Parik J, Wang Y, Papiha SS (2002). The emerging limbs and twigs of the East Asian mtDNA tree.. Mol Biol Evol.

[28] Kivisild T, Bamshad MJ, Kaldma K, Metspalu M, Metspalu E (1999). Deep common ancestry of Indian and western-Eurasian mitochondrial DNA lineages.. Curr Biol.

[29] Thangaraj K, Chaubey G, Kivisild T, Reddy AG, Singh VK (2005). Reconstructing the origin of Andaman Islanders.. Science.

[30] Macaulay V, Hill C, Achilli A, Rengo C, Clarke D (2005). Single, rapid coastal settlement of Asia revealed by analysis of complete mitochondrial genomes.. Science.

[31] Sun C, Kong QP, Palanichamy MG, Agrawal S, Bandelt HJ (2006). The Dazzling Array of Basal Branches in the mtDNA Macrohaplogroup M from India as Inferred from Complete Genomes.. Mol Biol Evol.

[32] Palanichamy MG, Sun C, Agrawal S, Bandelt HJ, Kong QP (2004). Phylogeny of mitochondrial DNA macrohaplogroup N in India, based on complete sequencing: implications for the peopling of South Asia.. Am J Hum Genet.

[33] Pierson MJ, Martinez-Arias R, Holland BR, Gemmell NJ, Hurles ME (2006). Deciphering Past Human Population Movements in Oceania: Provably Optimal Trees of 127 mtDNA Genomes.. Mol Biol Evol.

[34] Forster P, Torroni A, Renfrew C, Rohl A (2001). Phylogenetic star contraction applied to Asian and Papuan mtDNA evolution.. Mol Biol Evol.

[35] Friedlaender JS, Gentz F, Friedlaender F, Kaestle F, Schurr TG, Pawley A, Attenborough R, Golson J, Hyde R (2005). Mitochondrial genetic diversity and its determinants in Island Melanesia.. Papuan Pasts: Studies in the cultural, linguistic and biological history of the Papuan speaking peoples.

[36] Merriwether DA, Hodgson JA, Friedlaender FR, Allaby R, Cerchio S (2005). Ancient mitochondrial M haplogroups identified in the Southwest Pacific.. Proc Natl Acad Sci USA.

[37] Huoponen K, Schurr TG, Chen Y, Wallace DC (2001). Mitochondrial DNA variation in an aboriginal Australian population: evidence for genetic isolation and regional differentiation.. Hum Immunol.

[38] Cox MP (2003). Genetic Patterning at Austronesian Contact Zones [Ph. D.].

[39] Melton T, Clifford S, Martinson J, Batzer M, Stoneking M (1998). Genetic evidence for the proto-Austronesian homeland in Asia: mtDNA and nuclear DNA variation in Taiwanese aboriginal tribes.. Am J Hum Genet.

[40] Trejaut JA, Kivisild T, Loo JH, Lee CL, He CL (2005). Traces of Archaic Mitochondrial Lineages Persist in Austronesian-Speaking Formosan Populations.. Public Library of Science-Biology.

[41] Lum JK, Cann RL (1998). mtDNA and language support a common origin of Micronesians and Polynesians in Island Southeast Asia.. Am J Phys Anthropol.

[42] Cox MP (2005). Indonesian mitochondrial DNA and its opposition to a Pleistocene era origin of proto-Polynesians in island southeast Asia.. Hum Biol.

[43] Soodyall H, Jenkins T, Stoneking M (1995). ‘Polynesian’ mtDNA in the Malagasy.. Nat Genet.

[44] Merriwether DA, Friedlaender JS, Mediavilla J, Mgone C, Gentz F (1999). Mitochondrial DNA variation is an indicator of Austronesian influence in Island Melanesia.. Am J Phys Anthropol.

[45] Hill C, Soares P, Mormina M, Macaulay V, Clarke D (2007). A mitochondrial stratigraphy for island southeast Asia.. Am J Hum Genet.

[46] Jorde LB, Watkins WS, Bamshad MJ, Dixon ME, Ricker CE, Seielstad MT, Batzer MA (2000). The distribution of human genetic diversity: a comparison of mitochondrial, autosomal, and Y-chromosome data.. Am J Hum Genet.

[47] Wood ET, Stover DA, Ehret C, Destro-Bisol G, Spedini G (2005). Contrasting patterns of Y chromosome and mtDNA variation in Africa: evidence for sex-biased demographic processes.. Eur J Hum Genet.

[48] Lewis CM, Tito RY, Lizarraga B, Stone AC (2005). Land, language, and loci: mtDNA in Native Americans and the genetic history of Peru.. Am J Phys Anthropol.

[49] Rosenberg N, Pritchard J, Weber J, Cann H, Kidd K (2002). Genetic structure of human populations.. Science.

[50] Thurston WR, Dutton T, Tryon D (1994). Renovation and innovation in the languages of north-western New Britain.. Language contact and change in the Austronesian world.

[51] Friedlaender JS, Friedlaender FR, Hodgson J, McGrath S, Stoltz M, Friedlaender JS (2007). Mitochondrial DNA Variation in Northern Island Melanesia.. Genes, Language, and Culture Change in the Southwest Pacific.

[52] Friedlaender JS, Gentz F, Green K, Merriwether DA (2002). A cautionary tale on ancient migration detection: mitochondrial DNA variation in Santa Cruz Islands, Solomon Islands.. Hum Biol.

[53] Klopfstein S, Currat M, Excoffier L (2006). The fate of mutations surfing on the wave of a range expansion.. Mol Biol Evol.

[54] Ho SY, Phillips MJ, Cooper A, Drummond AJ (2005). Time Dependency of Molecular Rate Estimates and Systematic Overestimation of Recent Divergence Times.. Mol Biol Evol.

[55] Penny D (2005). Evolutionary biology: relativity for molecular clocks.. Nature.

[56] Edmonds CA, Lillie AS, Cavalli-Sforza LL (2004). Mutations arising in the wave front of an expanding population.. Proc Natl Acad Sci USA.

[57] Goodman M, Porter CA, Czelusniak J, Page SL, Schneider H (1998). Toward a phylogenetic classification of Primates based on DNA evidence complemented by fossil evidence.. Mol Phylogenet Evol.

[58] Kumar S, Filipski A, Swarna V, Walker A, Hedges SB (2005). Placing confidence limits on the molecular age of the human-chimpanzee divergence.. Proc Natl Acad Sci U S A.

[59] Bellwood P, Fox JJ, Tryon D, Bellwood P, Fox JJ, Tryon D (1995). The Austronesians in history: Common origins and diverse transformations.. The Austronesians: Historical and comparative perspectives.

[60] Oppenheimer S, Richards M (2001). Polynesian Origins: Slow boat to Melanesia?. Nature.

[61] Hagelberg E (1997). Ancient and modern mitochondrial DNA sequences and the colonization of the Pacific.. Electrophoresis.

[62] Hagelberg E, Clegg JB (1993). Genetic polymorphisms in prehistoric Pacific islanders determined by analysis of ancient bone DNA.. Proc Biol Sci.

[63] Ballinger SW, Schurr TG, Torroni A, Gan YY, Hodge JA (1992). Southeast Asian mitochondrial DNA analysis reveals genetic continuity of ancient Mongoloid migrations.. Genetics.

[64] Stoneking M (1990). Departure of human mitochondrial DNA variation from neutral expectations: An alternative explanation.. J Mol Evol.

[65] Rieder MJ, Taylor SL, Tobe VO, Nickerson DA (1998). Automating the identification of DNA variations using quality-based fluorescence re-sequencing: analysis of the human mitochondrial genome.. Nucleic Acids Res.

[66] Merriwether DA, Kaestle FA, Zemel B, Koki G, Mgone C, Surinder S, Papiha RD, Chakraborty Ranajit (1999). Mitochondrial DNA variation in the southwest Pacific.. Genomic diversity: Applications in human population genetics.

[67] Herrnstadt C, Elson JL, Fahy E, Preston G, Turnbull DM (2002). Reduced-median-network analysis of complete mitochondrial DNA coding-region sequences for the major African, Asian, and European haplogroups.. Am J Hum Genet.

[68] Trejaut JA, Loo J-H, Lin M (2006). Submitted (30-OCT-2006) Anthropological and Transfusion Medicine Research Laboratory, Mackay Memorial Hospital, 45, Min-sheng Road, Tamshui, Taipei County 251, Taiwan..

